# Management of acute decompensated cirrhosis

**DOI:** 10.1016/j.clinme.2025.100534

**Published:** 2025-11-20

**Authors:** Dina Mansour

**Affiliations:** a Department of Gastroenterology abd hepatology, Queen Elizabeth Hospital, Gateshead, NE9 6SX, UK; b Newcastle University, NE1 7RU, UK

**Keywords:** Cirrhosis, Liver, Decompensation, Ascites, Varices, Encephalopathy

## Abstract

Acute decompensated cirrhosis (AD) refers to the development of ascites, encephalopathy, gastrointestinal haemorrhage, or any combination of these disorders in a patient with known or previously undiagnosed advanced chronic liver disease. It carries a significant mortality, particularly if associated with organ failure (acute on chronic liver failure (ACLF)). Admissions with AD have increased by 50% over the last decade, and liver-related deaths have increased by 64% in the last 20 years. UK-wide reports and audits, including by the National Confidential Enquiry into Patient Outcome and Death, have revealed unwarranted variation in care and outcomes for patients with AD. This article summarises the management of patients admitted with AD, including the British Society of Gastroenterology (BSG) / British Association for the Study of the Liver (BASL) / Society of Acute Medicine (SAM) decompensated cirrhosis care bundle, developed for completion in the first 6 h of admission to standardise care for these complex patients.

## Highlights

• Acute decompensated cirrhosis (AD) refers to the development of ascites, hepatic encephalopathy, gastrointestinal haemorrhage, or any combination of these disorders in a patient with known, or previously undiagnosed, advanced liver disease.
• Acute decompensated cirrhosis carries a significant inpatient mortality (5-20%) and is frequently associated with complications including infection and acute kidney injury.
• The British Society of Gastroenterology (BSG) and British Association of the Study of the Liver (BASL) Decompensated Cirrhosis Admission Care Bundle summarises management of patients admitted with AD in the first 24 hours and should be completed in the emergency department or acute admissions unit within 6 hours of admission.
• Acute decompensated cirrhosis (AD) with organ failure is termed acute on chronic liver failure (ACLF), is associated with worse outcomes, and should prompt consideration of early escalation of care in appropriate patients, and parallel planning for patients not suitable for escalation.
• Ensuring appropriate follow up plans as included in the decompensated cirrhosis discharge bundle, including outpatient endoscopy, ultrasound surveillance, titration of diuretics, and early clinic review are key to reducing readmission rates.

## Introduction

Acute decompensated cirrhosis (AD) refers to the development of ascites, encephalopathy and/or gastrointestinal haemorrhage in patients with cirrhosis.^1^ It is associated with significant (5–20%) 28-day mortality.^2^

There has been a 50% increase in liver-related admissions in the last decade and a 64% increase in premature liver-related deaths in the last 20 years.^3^ Almost half of liver disease deaths occur in people of working age^4^ and liver disease mortality correlates with degree of social deprivation.^4^

Following the 2013 National Confidential Enquiry into Patient Outcome and Death (NCEPOD), which found that only 47% of patients admitted with AD received ‘good’ care,^5^ a decompensated cirrhosis care bundle was developed to guide management in the first 24 h of admission.^6^ This improved care,^7^ but the benefit was limited by low uptake (completed in only 11.4% of AD admissions in a nationwide audit).^8^

**Fig. 1:**
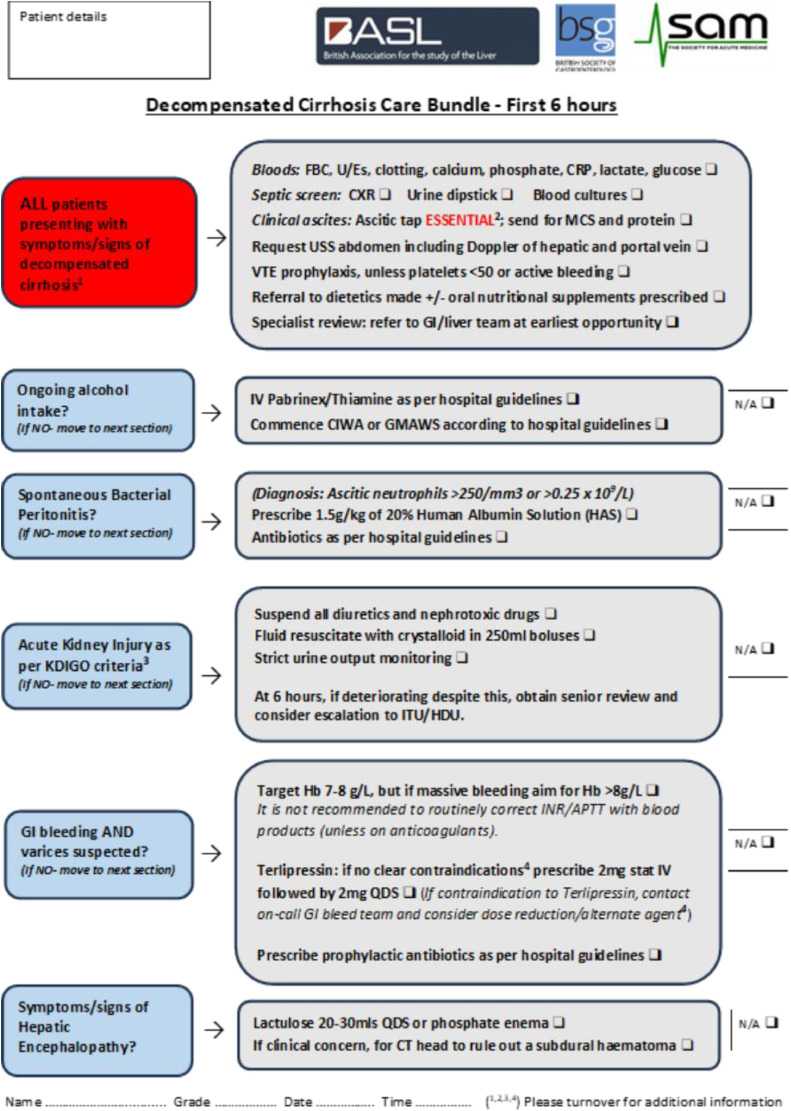
British Society of Gastroenterology, British Association for the Study of the Liver and Society of Acute Medicine Decompensated Cirrhosis Care Bundle – first 6 h.^9^

Following further recent NCEPOD review,^3^ the care bundle was revised (see Fig. 1)^9^ and strategies proposed to improve its use.^9^ It provides a structured approach to managing AD across the emergency department and admissions unit, and should be completed in all patients presenting with AD within the first 6 h of admission.

Patients admitted with AD should be cared for on a specialist gastroenterology or hepatology ward and should be seen by a specialist, ideally within 24 h of admission.

### History and examination

**Fig. 2:**
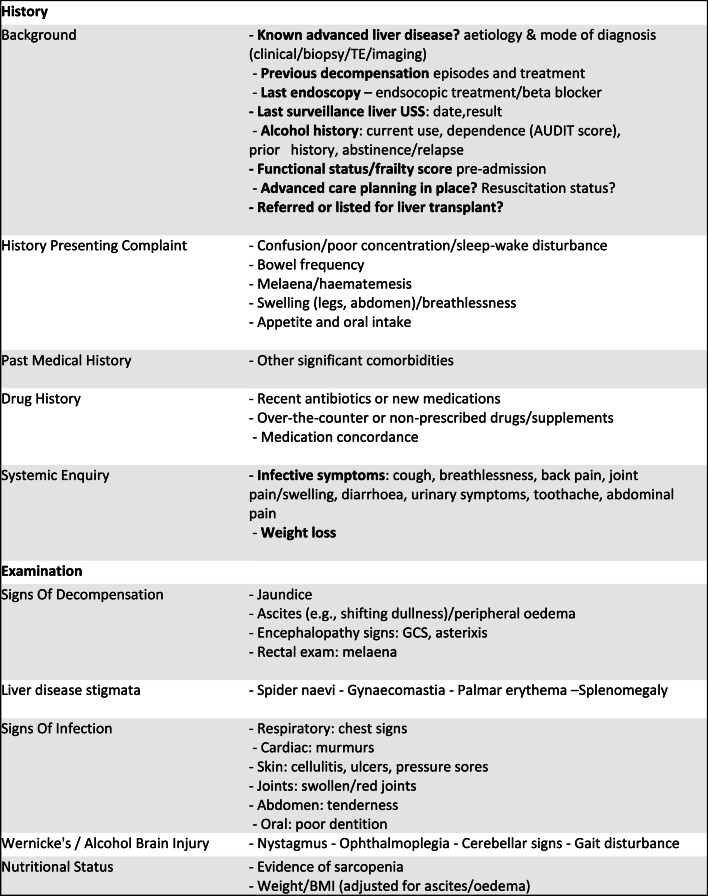
History and examination in patients presenting with acute decompensated.

A careful structured history and examination are important in helping to determine the stage of the patient’s disease, the potential precipitant of the decompensation, and to establish an appropriate ceiling of care. Key points in the history and examination from a patient with AD are summarised in Fig. 2.

### Investigations

Initial investigations and treatments are summarised in the care bundle^9^ (Fig. 1). A full non-invasive liver screen (including liver autoantibodies, ferritin and transferrin saturations, immunoglobulins, hepatitis B/C and HIV serology) is only required if not previously performed, but repeat viral hepatitis screen may be appropriate, including hepatitis A and E, particularly if liver enzymes are significantly raised.

A septic screen including an ascitic tap (sent for white cell/polymorph count (PMN) and culture, protein and albumin) should be performed to rule out infection, irrespective of inflammatory markers. Spontaneous bacterial peritonitis (SBP) occurs in approximately 10% of patients admitted with cirrhosis and is frequently asymptomatic, despite carrying a high mortality if treatment is delayed.^10^ Diagnosis is based on PMN cell count >250/mm^3^ or 0.25 × 10^9^/L ascitic fluid. Coagulopathy and/or thrombocytopenia are not a contraindication to an ascitic tap and checking/correction of coagulation is not required prior to the procedure.^11^

A Doppler ultrasound scan of the abdomen should be performed to look for hepatic/portal vein thrombosis, hepatocellular carcioma and ascites.

A CT head scan should be considered in patients with reduced consciousness or focal neurology, even if encephalopathy is suspected, particularly if there is a history of head injury or falls.

### Management

#### Alcohol

Patients with alcohol-related liver disease (ARLD) who continue to consume alcohol should be assessed for signs and symptoms of Wernicke’s encephalopathy (WE) and be prescribed parenteral thiamine (for at least 72 h in those with features of WE, or 24 h for those without features of WE)^9^ before stepping down to oral thiamine.^12^

Patients at risk of alcohol withdrawal should be assessed with a validated tool such as the Clinical Institute Withdrawal Assessment-Alcohol scale (CIWA)^13^ or the Glasgow Modified Assessment and Management of Alcohol tool (GMAWS)^14^ and treated with a symptom-triggered regimen of benzodiazepines. Chlordiazepoxide can accumulate in people with liver impairment/failure; consider using reduced doses or lorazepam (shorter acting and less likely to accumulate in patients with advanced liver disease) as an alternative or in combination.

All patients with alcohol dependence should be assessed by the alcohol care team and receive ongoing support with alcohol reduction/abstinence.^15^

#### Infections

Liver disease and alcohol both increase susceptibility to infections – patients may not display typical signs of infection or have significantly raised inflammatory markers, so a high index of suspicion is required.^16^ Multidrug-resistant organisms are a growing problem, therefore empirical antibiotics are not recommended.^1^ If suspicion of infection or signs of sepsis are present, a thorough history, examination and septic screen should be performed, followed by broad-spectrum antibiotics based on likely source in line with local guidelines, early de-escalation to narrow-spectrum antibiotics based on cultures, and cessation of antibiotics once infection is excluded.

Patients with suspected SBP should have prompt broad-spectrum antibiotics according to local guidelines, modified according to ascitic fluid culture results. SBP increases the risk of hepatorenal syndrome, therefore intravenous 20% albumin should be administered (1.5 g/kg albumin at diagnosis and 1 g/kg at 72 h).^1^ Routine albumin is not recommended in patients with other sources of infection.^17^

Patients treated for SBP should start secondary prophylaxis on completing their course of antibiotics, according to local protocol (eg cotrimoxazole 960 mg daily).^18^

#### Acute kidney injury (AKI)

AKI affects 20–30% of patients with AD, and it is associated with a poor outcome.^19^ The majority of AKI is pre-renal (47%) with 32% acute tubular necrosis (ATN) and 23% hepatorenal syndrome (HRS) (<1% post-renal).^20^

Diuretics, beta blockers and other nephrotoxic medication should be suspended and hypovolaemia corrected with crystalloids in 250 mL boluses, aiming for urine output (UO) >0.5 mL/kg/hr.^20^ Early treatment of infection is key. In patients with hypotension, UO <0.5 mL/kg/hr, worsening lactate or development of respiratory failure following 6 h of fluid resuscitation, consider escalation to a high dependency unit. Renal replacement therapy should be considered on a case-by-case basis – only approximately 25% recover following dialysis.^21^

HRS-AKI describes AKI associated with haemodynamic changes in patients with advanced liver disease and ascites. Without treatment, it carries a mortality approaching 100%.^10^ It is diagnosed in patients with AD and ascites, following volume expansion, treatment for any infection, and suspension of nephrotoxic medication, in the absence of structural renal disease (ie no proteinuria or haematuria, and normal kidneys on ultrasound imaging).^10^ It can co-exist with other causes of AKI. Treatment is with terlipressin (2–8 mg daily in divided doses or as continuous infusion, titrated to response) and 20% albumin 100 mL twice daily.^1^ Terlipressin should be used with caution in older patients (>70 years old), and patients with peripheral vascular disease or ischaemic heart disease (as it can cause ischaemia),^22^ as well as those with severe renal or liver impairment, due to increased risk of respiratory complications and sepsis.^23^

#### Acute variceal haemorrhage (AVH)

Acute upper gastrointestinal bleeding in people with known cirrhosis, or first presentation AD, should be treated as AVH until proven otherwise. AVH carries a 15% 30-day mortality.^24^ Resuscitation with blood according to the major haemorrhage protocol should be instituted in patients with haemodynamic instability and life-threatening bleed, aiming for a mean arterial pressure >65 mmHg (permissive hypotension may reduce rate of bleeding). A restrictive transfusion strategy is recommended in those who are haemodynamically stable (transfusion below 70 g/dL aiming for 70–80 g/dL).^25^ Platelet transfusion and fresh frozen plasma are not recommended outwith the major haemorrhage protocol, as this can increase portal hypertension and exacerbate bleeding.^25^ Tranexamic acid and proton pump inhibitors are of no benefit.^26^

Pre-endoscopy, terlipressin (2 mg then 1–2 mg four times a day, unless contraindications) and empirical intravenous antibiotics are recommended in AVH.^1^ In those with a contraindication to terlipressin, octreotide (50 μg followed by 50 μg/hr infusion) can be used.^27^ Recent evidence suggests that stopping terlipressin once haemostasis is confirmed (24 h post-endoscopic treatment with no evidence of bleeding) leads to better outcomes than continuing for 72 h.^28^ Intravenous erythromycin pre-endoscopy (250 mg) helps to clear blood from the stomach and improve views and endoscopic treatment.^29^

Endoscopy should be undertaken within 24 h of presentation.^29^ Prognostic scores such as the Blatchford score are not suitable for use in suspected portal hypertensive bleeding. Patients with active haematemesis, encephalopathy or haemodynamic instability should be intubated prior to endoscopy and discussed with critical care.^1^

### Hepatic encephalopathy (HE)

Hepatic encephalopathy can be precipitated by dehydration, electrolyte disturbance, infection, sedative medication and constipation. Ammonia levels are not required to diagnose patients with clinical HE, but a normal ammonia can be helpful in differentiating delirium from HE. All potential precipitants should be addressed by correcting electrolyte imbalance, suspending sedating medication, treating infections and by treating constipation with lactulose (aiming for 2–3 soft stools/day) and phosphate enemas if needed. Nasogastric tube placement should be considered in patients unable to take lactulose orally, and patients with reduced levels of consciousness (grade 3/4 encephalopathy) should be considered for escalation of care to critical care. Rifaximin can be added to those with recurrent/chronic encephalopathy to reduce risk of recurrence but does not acutely improve HE.^30^

### Ascites and oedema

Patients with ascites and/or oedema should have a moderately salt-restricted diet, under the supervision of a dietician.

Those with clinically tense ascites require paracentesis. In the absence of tense ascites, and/or where there is peripheral oedema, diuretics should be titrated every 72 h to aim for 0.5–1.0 kg weight loss per day. A combination of spironolactone (starting at 100 mg) and furosemide (starting at 40 mg) can be titrated according to potassium levels, renal function and response.^1^^,^^18^

### Nutrition

Malnutrition in AD is associated with increased complications including infections, HE and ascites, and mortality.^31^ Patients should eat every 2–3 h, including a bedtime snack, and have a high-protein diet (1.5 g/kg/day), rarely achievable in hospital without nutritional supplements.^31^ All patients should be reviewed by a dietician, and high protein supplements offered to those with/at risk of malnutrition or insufficient oral intake. Electrolytes (potassium, magnesium and phosphate) should be monitored and replaced if depleted. Enteral feeding should be considered in those who are unable to tolerate sufficient calories orally, but can be risky in very confused/agitated patients.

### VTE prophylaxis

Clotting parameters in patients with cirrhosis do not accurately reflect bleeding risk, and patients with AD are at increased risk of thrombosis, so all patients with platelet count >50 should have tinzaparin thromboprophylaxis in the absence of active bleeding.^9^

### Alcohol-associated hepatitis (AH)

Alcohol-associated hepatitis is a clinical syndrome characterised by acute-onset jaundice in the setting of long-term heavy alcohol use. Laboratory-based prognostic scores help determine disease severity and guide management.^32^ The most important determinant of long-term outcome is abstinence from alcohol – optimising nutrition, treating concomitant infections and supportive treatment for complications (including ascites, HE, AVH, AKI) are the mainstay of in-hospital treatment.^33^

Corticosteroids (prednisolone 40 mg od) are thought to be beneficial for a subgroup of patients with severe AH.^32^ Infection and bleeding should be excluded prior to starting steroids. If bilirubin starts to fall in this period, corticosteroids may not be required. If corticosteroids are started, response should be assessed at day 4–7 using the Lille score,^34^^,^^35^ and treatment stopped in those who are not responding.^32^ In those who respond, prednisolone is continued for 28 days.

### The deteriorating patient

Patients with AD who develop acute on chronic liver failure (ACLF), a severe form of decompensation involving organ failure and systemic inflammation, and are suitable for treatment escalation should be referred to critical care.^2^ Patients under 60 years old with no contraindications to transplant and, in the case of ARLD, abstinent for >3 months who develop three-organ failure can now be referred for consideration of urgent liver transplant assessment. A UK pilot demonstrated that transplant prioritisation improved survival for this group.^20^

In patients who deteriorate despite treatment, and in whom escalation is considered futile, early palliative care should be considered. Palliative care in advanced liver disease is covered elsewhere and is beyond the scope of this article. However, advanced liver disease carries a significant physical and psychosocial burden, particularly in the last year of life, and parallel planning with early palliative care improves quality of life and even prognosis.^36^

### Discharge

Patients admitted with alcohol-related liver disease (AD) have high readmission rates. Ensuring appropriate follow-up on discharge is essential. Patients should receive clear self-management advice, contact details for the liver team, and support for alcohol abstinence where needed.

**Fig. 3:**
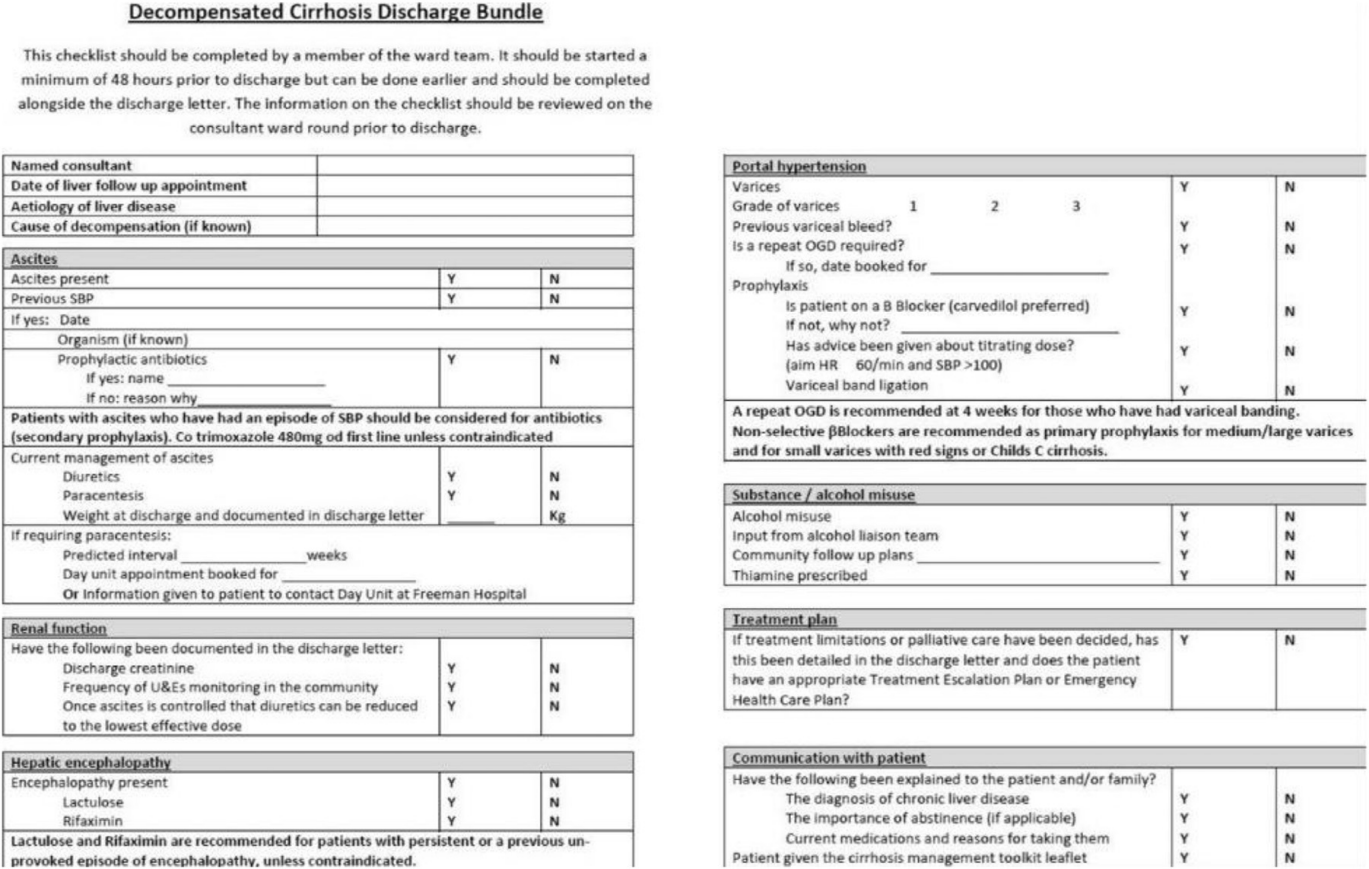
Decompensated cirrhosis discharge bundle.^37^

Use of a cirrhosis discharge bundle (Fig. 3), has been shown to improve follow-up and reduce readmissions.^37^ Early discharge clinics can also reduce readmissions and length of stay, and improve outcomes.^38^

Advanced care planning should also be considered, discussed with the patient and family, shared with primary care, and include community palliative input where ongoing support is required.

## Funding

This research did not receive any specific grant from funding agencies in the public, commercial or not-for-profit sectors.

## CRediT authorship contribution statement

**Dina Mansour:** Writing – review & editing, Writing – original draft, Conceptualization.

## Declaration of competing interest

The authors declare that they have no known competing financial interests or personal relationships that could have appeared to influence the work reported in this paper.

## References

[1] Angeli P., Bernardi M., Villanueva C. (2018). EASL clinical practice guidelines for the management of patients with decompensated cirrhosis. J Hepatol.

[2] Moreau R., Tonon M., Krag A. (2023). EASL clinical practice guidelines on acute-on-chronic liver failure. J Hepatol.

[3] NCEPOD (2022). Remeasuring the units: an update on the organisation of alcohol-related liver disease services. National Confidential Enquiry into Patient Outcome and. Death.

[4] (2022). Office for Health Improvement & Disparities. Liver disease: applying all our health.

[5] NCEPOD (2013).

[6] McPherson S., Dyson J., Austin A., Hudson M. (2016). Response to the NCEPOD report: development of a care bundle for patients admitted with decompensated cirrhosis – the first 24 h. Frontline Gastroenterol.

[7] Dyson J.K., Rajasekhar P., Wetten A. (2016). Implementation of a ‘care bundle’ improves the management of patients admitted to hospital with decompensated cirrhosis. Aliment Pharmacol Ther.

[8] Trainee Collaborative for Research and Audit in Hepatology UK (2024). Admission care bundles for decompensated cirrhosis are poorly utilised across the UK: results from a multi-centre retrospective study. Clin Med.

[9] McPherson S., Abbas N., Allison M.E.D. (2025). Decompensated cirrhosis: an update of the BSG/BASL admission care bundle. Frontline Gastroenterol.

[10] Biggins S.W., Angeli P., Garcia-Tsao G. (2021). Diagnosis, evaluation, and management of ascites, spontaneous bacterial peritonitis and hepatorenal syndrome: 2021 practice guidance by the American association for the study of liver diseases. Hepatology.

[11] Mansour D., Masson S., Corless L. (2023). British society of gastroenterology best practice guidance: outpatient management of cirrhosis–part 2: decompensated cirrhosis. Frontline Gastroenterol.

[12] NICE (2010). NICE clinical guideline 100: alcohol-use disorders: diagnosis and clinical management of alcohol-related physical complications.

[13] Sullivan J.T., Sykora K., Schneiderman J., Naranjo C.A., Sellers EM. (1989). Assessment of alcohol withdrawal: the revised clinical institute withdrawal assessment for alcohol scale (CIWA-Ar). Br J Addict.

[14] McPherson A., Benson G., Forrest EH. (2012). Appraisal of the Glasgow assessment and management of alcohol guideline: a comprehensive alcohol management protocol for use in general hospitals. QJM.

[15] Moriarty KJ. (2020). Alcohol care teams: where are we now?. Frontline Gastroenterol.

[16] Piano S., Singh V., Caraceni P. (2019). Epidemiology and effects of bacterial infections in patients with cirrhosis worldwide. Gastroenterology.

[17] China L., Freemantle N., Forrest E. (2021). A randomized trial of albumin infusions in hospitalized patients with cirrhosis. N Engl J Med.

[18] Aithal G.P., Palaniyappan N., China L. (2021). Guidelines on the management of ascites in cirrhosis. Gut.

[19] Tariq R., Hadi Y., Chahal K., Reddy S., Salameh H., Singal AK. (2020). Incidence, mortality and predictors of acute kidney injury in patients with cirrhosis: a systematic review and meta-analysis. J Clin Transl Hepatol.

[20] Garcia-Tsao G., Parikh C.R., Viola A. (2008). Acute kidney injury in cirrhosis. Hepatology.

[21] Wang P.L., Silver S.A., Djerboua M., Thanabalasingam S., Zarnke S., Flemming JA. (2022). Recovery from dialysis-treated acute kidney injury in patients with cirrhosis: a population-based study. Am J Kidney Dis.

[22] Gluud L.L., Christensen K., Christensen E., Krag A. (2010). Systematic review of randomized trials on vasoconstrictor drugs for hepatorenal syndrome. Hepatology.

[23] MHRA. Terlipressin: new recommendations to reduce risks of respiratory failure and septic shock in patients with type 1 hepatorenal syndrome. 2024. Available: https://www.gov.uk/drug-safety-update/terlipressin-new-recommendations-to-reduce-risks-of-respiratory-failure-and-septic-shock-in-patients-with-type-1-hepatorenal-syndrome.

[24] Jairath V., Rehal S., Logan R. (2014). Acute variceal haemorrhage in the united kingdom: patient characteristics, management and outcomes in a nationwide audit. Dig Liver Dis.

[25] Villanueva C., Colomo A., Bosch A. (2013). Transfusion strategies for acute upper gastrointestinal bleeding. N Engl J Med.

[26] Roberts I., Shakur-Still H., Afolabi A. (2020). Effects of a high-dose 24-h infusion of tranexamic acid on death and thromboembolic events in patients with acute gastrointestinal bleeding (HALT-IT): an international randomised, double-blind, placebo-controlled trial. Lancet.

[27] Sridharan K., Sivaramakrishnan G. (2019). Vasoactive agents for the management of variceal bleeding: a mixed treatment comparison network meta-analysis and trial sequential analysis of randomized clinical trials. Drug Res.

[28] Vaishnav M., Biswas S., Shenoy A. (2024). Comparison of 1-day versus 3-day intravenous terlipressin in cirrhosis patients with variceal bleeding: a pilot randomised controlled trial. Aliment Pharmacol Ther.

[29] Siau K., Hearnshaw S., Stanley A.J. (2020). British society of gastroenterology (BSG)-led multisociety consensus care bundle for the early clinical management of acute upper gastrointestinal bleeding. Frontline Gastroenterol.

[30] Montagnese S., Rautou P., Romero-Gómez M. (2022). EASL clinical practice guidelines on the management of hepatic encephalopathy. J Hepatol.

[31] Merli M., Berzigotti A., Zelber-Sagi S. (2019). EASL clinical practice guidelines on nutrition in chronic liver disease. J Hepatol.

[32] Parker R., Allison M., Anderson S. (2023). Quality standards for the management of alcohol-related liver disease: consensus recommendations from the British Association for the study of the liver and British Society of Gastroenterology ARLD Special Interest Group. BMJ Open Gastro.

[33] Thursz M., Gual A., Lackner C. (2018). EASL clinical practice guidelines: management of alcohol-related liver disease. J Hepatol.

[34] Garcia-Saenz-de-Sicilia M., Duvoor C., Altamirano J. (2017). A day-4 Lille model predicts response to corticosteroids and mortality in severe alcoholic hepatitis. Am J Gastroenterol.

[35] Louvet A., Naveau S., Abdelnour M. (2007). The Lille model: a new tool for therapeutic strategy in patients with severe alcoholic hepatitis treated with steroids. Hepatology.

[36] Woodland H., Hudson B., Forbes K., McCune A., Wright M. (2020). Palliative care in liver disease: what does good look like?. Frontline Gastroenterol.

[37] Smethurst K., Gallacher J., Jopson L. (2022). Improved outcomes following the implementation of a decompensated cirrhosis discharge bundle. Frontline Gastroenterol.

[38] Giles B., Fancey K., Gamble K. (2024). Novel, nurse-led early postdischarge clinic is associated with fewer readmissions and lower mortality following hospitalisation with decompensated cirrhosis. Frontline Gastroenterol.

